# Seasonal and age-related variations impacting HPV vaccination uptake in Romania

**DOI:** 10.1186/1471-2334-13-S1-P28

**Published:** 2013-12-16

**Authors:** Oana Streinu-Cercel, Anca Streinu-Cercel, Alexandru Rafila, Adrian Pană, Adriana Pistol, Mihai Săndulescu, Adrian Streinu-Cercel

**Affiliations:** 1 Carol Davila University of Medicine and Pharmacy, Bucharest, Romania; 2 National Institute for Infectious Diseases “Prof. Dr. Matei Balş”, Bucharest, Romania; 3 Romanian Ministry of Health, Bucharest, Romania; 4 National Institute of Public Health, Bucharest, Romania

## Background

We report data regarding the influence of seasonal factors and patient age on the uptake of HPV vaccination in Romania, following the introduction of the vaccine in the national immunization schedule.

## Methods

We prospectively collected data regarding the national immunization schedule between June 2010 and June 2013. For this report, we analyzed the impact of age and seasonal variations on the number of vaccines administered monthly.

## Results

In the studied time span 80,509 HPV vaccine doses were administered in Romania, 46% (37,359) of these representing the third and final dose of the vaccination regimen. By the end of June 2013, 38,281 women of all ages had completed the full 3-dose HPV vaccination regimen.

The studied time span appears to show a biphasic evolution of the vaccination schedule: between June 2010 and August 2011, absolute numbers were constantly above 1,500 doses per month, while after August 2011 there was an important decrease, with the total number of shots per months constantly below 1,000 but above 150.

The months with the lowest numbers reported were April (2,787), June (2,196) and July (1,649) 2011 in the first time span and September (250), October (245) 2011, September 2012 (186), February (227) and March (205) 2013 for the second time span.

The number of girls undergoing vaccination during June-December 2010 was higher in the lower age groups, with 10,456 girls in the 12-14 years old age group, 13,135 (15-19 years old), 15,723 (20-24 years old), 10,785 (age 24 and over). After 2011, the proportions changed, with the largest numbers of vaccines being administered at increasingly higher ages: 1,671 (12-14 years old), 4,737 (15-19 years old), 8,152 (20-24 years old), 9,800 (age 24 and over) during 2011; 319 (12-14 years old), 598 (15-19 years old), 1,585 (20-24 years old), 1,821 (age 24 and over) during 2012 and 120 (12-14 years old), 215 (15-19 years old), 518 (20-24 years old), 874 (age 24 and over) during January-June 2013.

## Conclusion

Romania has recorded an interesting shift from the initial school vaccination campaigns to the more recent uptake of vaccine in women of all ages.

